# The Cell Biology of Tau Secretion

**DOI:** 10.3389/fnmol.2020.569818

**Published:** 2020-09-23

**Authors:** Maria Merezhko, Riikka-Liisa Uronen, Henri J. Huttunen

**Affiliations:** Neuroscience Center, HiLIFE, University of Helsinki, Helsinki, Finland

**Keywords:** tau, unconventional protein secretion, membranous organelles-based secretion, extracellular vesicles, autophagy, lysosomes, synaptic transmission, glial cells

## Abstract

The progressive accumulation and spread of misfolded tau protein in the nervous system is the hallmark of tauopathies, progressive neurodegenerative diseases with only symptomatic treatments available. A growing body of evidence suggests that spreading of tau pathology can occur *via* cell-to-cell transfer involving secretion and internalization of pathological forms of tau protein followed by templated misfolding of normal tau in recipient cells. Several studies have addressed the cell biological mechanisms of tau secretion. It now appears that instead of a single mechanism, cells can secrete tau *via* three coexisting pathways: (1) translocation through the plasma membrane; (2) membranous organelles-based secretion; and (3) ectosomal shedding. The relative importance of these pathways in the secretion of normal and pathological tau is still elusive, though. Moreover, glial cells contribute to tau propagation, and the involvement of different cell types, as well as different secretion pathways, complicates the understanding of prion-like propagation of tauopathy. One of the important regulators of tau secretion in neuronal activity, but its mechanistic connection to tau secretion remains unclear and may involve all three secretion pathways of tau. This review article summarizes recent advancements in the field of tau secretion with an emphasis on cell biological aspects of the secretion process and discusses the role of neuronal activity and glial cells in the spread of pathological forms of tau.

## Introduction

Neurodegenerative diseases are incurable and disabling conditions characterized by progressive degeneration and loss of cells, structures, and functions of the nervous system. Although clinically neurodegenerative disorders (NDDs) have a broad range of manifestations, generally they cause progressive cognitive and motor dysfunctions (Gan et al., 2018). The majority of NDDs are sporadic, but hereditary conditions also exist; the examples of the latter include Huntington’s disease (HD) and spinocerebellar ataxias. Many diseases that are predominantly sporadic with the multifactorial origin, such as Alzheimer’s disease (AD) and Parkinson’s disease (PD), also have familial forms, typically indistinguishable from the sporadic diseases in their clinical manifestations and neuropathology (Lippa et al., 1996; Papapetropoulos et al., 2007). Classification of NDDs is often done neuropathologically based on specific proteins that form insoluble protein deposits in neurons and/or glia. Diseases with an accumulation of tau aggregates are termed tauopathies and include AD, progressive supranuclear palsy (PSP), corticobasal degeneration (CBD), frontotemporal dementia and parkinsonism linked to chromosome 17 (FTDP-17), argyrophilic grain disease (AGD), aging-related tau astrogliopathy (ARTAG) and primary age-related tauopathy (PART; Arendt et al., 2016). Tau aggregates and other disease-characteristic protein deposits, however, are not restricted to the associated clinical profiles—they often coexist in individual patients or can occur in non-diseased individuals (Spires-Jones et al., 2017; Yan et al., 2020).

The common feature of multiple neurodegenerative diseases, including tauopathies, is the progressive accumulation of misfolded proteins in the nervous system. These misfolded proteins or their aggregated forms can be transmitted from affected cells to healthy cells where they can induce templated misfolding and pathological aggregation of the same type of protein. This cell-to-cell transmission of pathologically misfolded and aggregated protein species is now thought to form the mechanistic basis of disease progression in NDDs. Interestingly, while the propagation of misfolded protein pathology appears to be a common mechanism shared by various NDDs, many NDDs are characterized by the aggregation of specific proteins in distinctive neuroanatomical patterns and locations in the central nervous system (CNS) and/or in the peripheral nervous system (Jucker and Walker, 2013). This review article will address the mechanisms of tau secretion, the first step in the transcellular propagation of tau pathology.

## Tau Protein in Health and Disease

Tau is one of the major microtubule-associated proteins (MAPs) in neurons whose main role is to stabilize microtubules, supporting cytoskeletal organization, and axonal transport (Barbier et al., 2019). Recent studies, however, suggest that instead of stabilizing microtubules, tau may enable them to have a long labile domain, microtubule fraction with rapid dynamics (Qiang et al., 2018). Dynamic interaction between tau and microtubules regulates multiple cellular functions, including neurite polarity and stability, motor-driven axonal transport of vesicles and organelles, and, outgrowth, elongation, and guidance of axons (Caceres and Kosik, 1990; Esmaeli-Azad et al., 1994; Kempf et al., 1996; Takei et al., 2000; Dixit et al., 2008; Li et al., 2014; Biswas and Kalil, 2018; Tapia-Rojas et al., 2019). In addition to its functions as a MAP, tau also plays a role in DNA protection, adult neurogenesis, synaptic plasticity, regulation of neuronal activity, and insulin signaling (Hong et al., 2010; Sultan et al., 2011; DeVos et al., 2013; Kimura et al., 2014; Marciniak et al., 2017). Besides neurons, tau is also expressed at low levels in oligodendrocytes and possibly astrocytes (Müller et al., 1997; Seiberlich et al., 2015; Kovacs, 2020).

The tau protein is composed of four structurally and functionally distinct domains: (1) N-terminal projection domain; (2) the central proline-rich domain; (3) microtubule-binding repeat domain (MTBD); and (4) C-terminal projection domain (Guo et al., 2017). The primary function of the MTBD, consisting of three or four imperfectly repeated motifs, is binding to microtubules (Guo et al., 2017). MTBD can also bind to actin and crosslink it with microtubules (Cabrales Fontela et al., 2017). The N-terminal projection domain regulates the binding of tau to microtubules and determines spacings between them (Chen et al., 1992; Derisbourg et al., 2015). The proline-rich region is involved in cell signaling, binding to actin and tubulin (He et al., 2009; Arendt et al., 2016). A recent study suggested that the proline-rich region binds tubulin stronger than MTBD and may have a primary role in microtubule polymerization (McKibben and Rhoades, 2019). The C-terminal projection domain also contributes to binding of tau to tubulin and regulates tau binding to the plasma membrane (Arendt et al., 2016; Kadavath et al., 2018). As the structure and physiological functions of tau are beyond the scope of the current review, the readers are referred to excellent reviews on these topics (Arendt et al., 2016; Guo et al., 2017; Tapia-Rojas et al., 2019).

The structure of tau is crucial for its functions, but as tau belongs to a group of intrinsically disordered proteins (IDPs), it has no stable sequence-defined secondary structure in solution (Jeganathan et al., 2008). Instead, tau can rapidly adopt multiple conformations upon interaction with multiple binding partners, including other proteins, small molecules, membranes, and nucleic acids (Jeganathan et al., 2008; Georgieva et al., 2014; Qi et al., 2015; Rauch et al., 2017). Free tau protein, however, is not entirely in a random coil form. Instead, it tends to adopt a compact paperclip-like or S-shaped conformation, which protects tau from aggregation by masking the regions involved in it (Jeganathan et al., 2006; Elbaum-Garfinkle and Rhoades, 2012; Zhu et al., 2015). Being an IDP, tau is easily accessible to post-translational modifications, and a large and diverse set of modifications (e.g., phosphorylation, acetylation, methylation, ubiquitination, sumoylation, glycosylation, and O-GlcNAcylation) are known to regulate tau function (Martin et al., 2011; Owen and Shewmaker, 2019). Of all modifications, phosphorylation has a particular importance, as abnormal phosphorylation of tau strongly contributes to tau aggregation (Alonso et al., 2001; Zhu et al., 2015). Although, a physiological cycle of phosphorylation and dephosphorylation is necessary to maintain biological functions of tau, in pathological conditions a level of phosphorylation at specific disease-associated sites increases, reducing tau’s affinity to microtubules (Biernat et al., 1993; Bramblett et al., 1993; Sengupta et al., 1998; Cho and Johnson, 2004; Barbier et al., 2019).

Eventually, pathologically phosphorylated (or hyperphosphorylated) tau detaches from microtubules, destabilizing the cytoskeleton and compromising axonal transport (Barbier et al., 2019; Combs et al., 2019). Most importantly, the accumulating hyperphosphorylated tau in the cytosol may misfold resulting in the formation of tau aggregates and fibrils, which are the most prominent hallmarks of tauopathies (Arendt et al., 2016). Although the tau aggregation pathway, as well as its filament structure and composition, are not universal but specific to each tauopathy, in general, it involves the following steps: (1) acquiring aggregation-competent conformation that differs from the paperclip-like conformation of physiological tau in solution; (2) formation of dimers and small soluble oligomers (pre-tangles); and (3) formation of filamentous inclusions (Sanders et al., 2014; Falcon et al., 2018, 2019; Cieplak, 2019). The large insoluble inclusions of tau, however, are not the most toxic tau species. They may protect cells from damage by the very toxic small soluble oligomers of tau through segregating them from the cell environment into large insoluble inclusions (Cowan and Mudher, 2013; d’Orange et al., 2018). Besides the intracellular toxicity of a certain tau species, their ability to induce damage also depends on the ability of certain tau species to affect neighboring cells and propagate the pathology further.

## The Tau Propagation Concept

The stereotypical appearance and progression of tau pathology differ considerably between tauopathies—the pathology starts in distinct anatomical areas and in some cases may involve glial cells in addition to neurons (Braak and Braak, 1995; Williams et al., 2007; Irwin et al., 2016). Nevertheless, in all tauopathies, the spread of tau pathology correlates with the progression of the disease and can be used for their neuropathological staging. For instance, in AD tau pathology first appears in the transentorhinal cortex in the medial temporal lobe (Braak stages I/II, or preclinical AD), then it progresses to limbic regions (Braak stages III/IV, or prodromal AD) and finally to the neocortex (Braak stages V/VI, or clinical AD; Braak and Braak, 1995).

In Pick’s disease, tau pathology affects mainly neurons and to a lesser extent glial cells. Tau-positive inclusions first appear in frontotemporal limbic/paralimbic and neocortical regions, then progress to subcortical structures, followed by the primary motor cortex and precerebellar nuclei, and finally by the visual cortex (Irwin et al., 2016). In PSP, tau pathology affects both neuronal and glial cells, starting from the subthalamic nucleus, pallidum, and substantia nigra, then progressing to the pedunculopontine nucleus, dentate nucleus, and frontal lobe, and finally to the temporal lobe (Williams et al., 2007). Tau pathology in AGD initially develops at the ambient gyrus, then progresses to the anterior and posterior medial temporal lobe, and finally affects the septum, insular cortex, and anterior cingulate gyrus (Saito et al., 2004). Despite the differences in the stereotypical neuropathological patterns in these disorders, the pathology in tauopathies, as well as in other NDDs, seems to spread along neuroanatomically connected brain areas (Raj et al., 2012; Pandya et al., 2017). The hypothesis of prion-like propagation offers an elegant explanation of this phenomenon (Mudher et al., 2017).

Prions are infectious misfolded proteins that can move from cell to cell and transmit their misfolded conformation, “a prion template,” to their native “healthy” versions in the previously unaffected cells, thus propagating the pathology in a template-directed manner (Sigurdson et al., 2019). Many proteins involved in NDDs, including Aβ, α-synuclein (α-syn) and tau, although they are not prions themselves, possess prion-like properties. This means that certain misfolded forms of these proteins can be transmitted from a diseased neuron to an unaffected neuron, where they can induce further misfolding and aggregation of the native forms of the protein (Jucker and Walker, 2018).

Indeed, numerous studies have demonstrated that extracts from diseased human or mouse brain transferred to healthy cells, intracerebrally injected or even peripherally administrated to wild-type mice or mouse disease models, can seed and propagate the source pathology in a prion-like manner (Kane et al., 2000; Clavaguera et al., 2009, 2014; Lasagna-Reeves et al., 2012; Luk et al., 2012). Furthermore, grafting of dura matter infected with prions causing iatrogenic Creutzfeldt-Jakob disease (iCJD), which frequently associates with Aβ deposition, provided an opportunity to study graft to host transmission of Aβ in the human brain (Frontzek et al., 2016; Hamaguchi et al., 2016). Additionally, post mortem analysis of brains from Parkinson’s and HD patients who had received embryonic tissue grafts revealed transmission in the opposite direction, from host to graft, of α-syn, huntingtin protein and tau (Kordower et al., 2008; Cicchetti et al., 2014; Cisbani et al., 2017). Finally, an examination of several cases of iCJD that appears to be linked to the treatment in childhood with human tissue-derived growth hormone, which may have contained CJD prions and Aβ, suggested that Aβ may also traffic from the periphery to the brain and induce Aβ pathology (Ritchie et al., 2017; Purro et al., 2018).

## The Mechanism of Tau Propagation

The prion-like propagation of tauopathies involves the following steps: (1) generation of a seed, a tau unit able to propagate the pathology; (2) secretion of seed-competent tau from a diseased cell; (3) uptake of seed-competent tau by an unaffected cell; and (4) templated misfolding generating new tau seeds in the recipient cell.

For tau, a single misfolded protein in a specific conformation can seed aggregation (Michel et al., 2014; Mirbaha et al., 2018; Sharma et al., 2018). Seed-competent tau monomers have been reported to form morphologically distinct “strains” in different tauopathies, meaning that a conformation of tau in a given seed will define the distinct type of inclusions, rate of spread, and pattern of the neuropathological lesion in each disease (Sanders et al., 2014; Kaufman et al., 2016; Narasimhan et al., 2017; Sharma et al., 2018). All these properties can be replicated in the process of cell-to-cell transmission *in vitro* and *in vivo*. Indeed, numerous studies have demonstrated that intracranial injections of tau strains derived from distinct tauopathies into healthy wild-type animals resulted in the development of patterns of tau pathology similar to the source tauopathy (Clavaguera et al., 2013; Sanders et al., 2014; Boluda et al., 2015; Kaufman et al., 2016; Narasimhan et al., 2017). Additionally, exposure of cultured cells to distinct tau strains resulted in the formation of morphologically similar tau inclusions as in the source tauopathy, and this process could be faithfully replicated *in vivo* over many generations of mice (Sanders et al., 2014). Interestingly, while tau in AD forms a single strain, tau in CBD forms three sub-strains and monomers from any of these strains can induce the formation of all three sub-strains when inoculated into naïve cells (Sharma et al., 2018).

Pathogenic seeds need to exit donor cells for tau pathology to propagate. They can follow one of the several potential secretion pathways to reach the extracellular. The possible mechanisms of tau secretion include: (1) translocation through the plasma membrane; (2) membranous organelle-based secretion (MOBS); and (3) ectosomal secretion (Lonati et al., 2018; Kang et al., 2019; Pernègre et al., 2019). These secretion mechanisms of tau are reviewed in more detail in the following sections.

Tau does not spread randomly but appears to follow a disease-specific spatiotemporal pattern based on anatomically connected neuronal networks, meaning that a presynaptic neuron secretes tau and a post-synaptic neuron takes it up. Neuronal activity may play an important role in the trans-synaptic transfer, as it has been demonstrated to stimulate both tau secretion by neurons and tau propagation in tissue (Pooler et al., 2013; Yamada et al., 2014; Wu et al., 2016). The activity-stimulated trans-synaptic transfer, however, may not be the only way of cell-to-cell transmission of tau, as the process is known to occur also in a retrograde direction along a neuronal network. Thus, interstitial diffusion or microglia-assisted mechanisms also likely contribute to the propagation process (Ahmed et al., 2014; Asai et al., 2015; Takeda et al., 2015).

Secreted tau reaches the surface of a recipient neuron in either free naked form or inside of extracellular vesicles. Recent studies have shown that both aggregated and monomeric free tau can be internalized by rapid dynamin-dependent endocytosis, independent of clathrin, while monomeric tau can also undergo slower actin-dependent macropinocytosis (Holmes et al., 2013; Wu et al., 2013; Evans et al., 2018). M1/M3 muscarinic receptor and low-density lipoprotein receptor-related protein 1 (LRP1) may serve as receptors that trigger tau endocytosis (Morozova et al., 2019; Rauch et al., 2020). Cell-surface heparan sulfate proteoglycans (HSPGs), e.g., syndecans, also facilitate endocytosis by recruiting tau molecules to the plasma membrane (Holmes et al., 2013; Rauch et al., 2018; Hudák et al., 2019; Zhao et al., 2020). In both internalization pathways, tau enters the cytosol of recipient neurons inside vesicular structures, either endosomes or macropinosomes, but later it escapes these vesicles, possibly by permeabilization of the endosome membrane to get access to native tau in the cytosol to initiate templated misfolding (Calafate et al., 2016).

Tau secreted inside vesicles, such as exosomes, undergoes uptake by recipient cells as well (Polanco et al., 2016; Wang et al., 2017). Although these pathways have not been specifically demonstrated for tau internalization, cells can internalize extracellular vesicles by multiple pathways, including clathrin-dependent and clathrin-independent endocytosis, macropinocytosis, phagocytosis, cholesterol/sphingomyelin rich domains-mediated internalization, and membrane fusion (Mulcahy et al., 2014).

Besides secretion and uptake, tau transfer can occur *via* tunneling nanotubes, filamentous actin-containing membranous structures that transiently connect cytosols of cells and mediate the selective transfer of organelles, vesicles, proteins, and small molecules between connected cells (Abounit et al., 2016b; Tardivel et al., 2016; Ariazi et al., 2017). Furthermore, tau and other prion-like proteins implicated in neurodegenerative diseases do not only use tunneling nanotubes for transfer but also promote their formation and, therefore, their transfer between cells (Costanzo et al., 2013; Abounit et al., 2016a,b; Tardivel et al., 2016; Rostami et al., 2017). In theory, tau can be transferred between cells through the tunneling nanotubes both as a free protein or inside of vesicular structures such as late endosomes (LE)/lysosomes. Readers interested in more detailed reviews in the field of tau propagation are referred to the following insightful articles (Goedert et al., 2017; Mudher et al., 2017; Jucker and Walker, 2018; Gibbons et al., 2019; Peng et al., 2020).

## Tau Secretion

Proteins can be secreted to the extracellular space *via* multiple mechanisms classified as either conventional/classical or unconventional pathways. The vast majority of secreted proteins follow a well-studied conventional secretory route, also known as the endoplasmic reticulum (ER)-Golgi pathway. This pathway is devoted to proteins that contain a signal peptide (also known as a leader sequence), generally at the N-terminus.

Proteins without a signal peptide (also called leaderless proteins) can follow one of four unconventional secretory pathways: (1) direct translocation through the plasma membrane through a self-made pore; (2) ATP-binding cassette (ABC) transporter-based secretion; (3) membranous organelles-based unconventional secretion (MOBS); and (4) microvesicle shedding at the plasma membrane (Nickel and Rabouille, 2009; Rabouille, 2017; Lee and Ye, 2018). While the first two pathways always result in the secretion of a protein to the extracellular space in a free form, non-bound to any vesicles, the third pathway may result in the secretion of either free protein or a protein inside the small vesicles called exosomes. The fourth pathway always results in the secretion of a protein inside large membrane-derived vesicles, called ectosomes. A common feature of the unconventional protein secretion pathways is that in most cases they are induced by various types of cellular stress (Giuliani et al., 2011; Kim et al., 2018).

**Figure 1:**
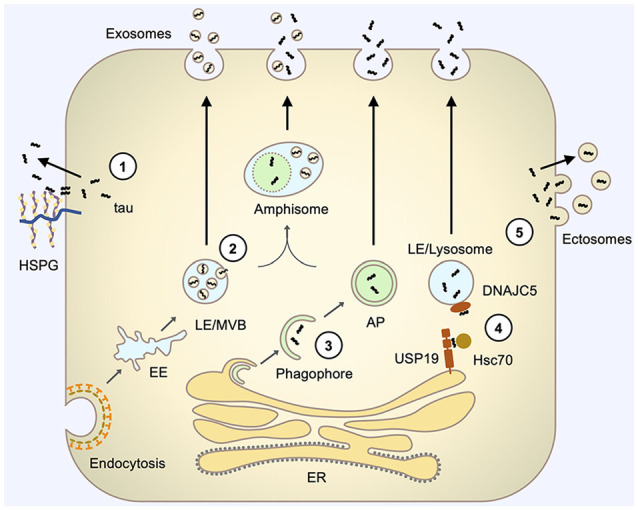
Pathways involved in tau secretion. (1) Direct translocation through the plasma membrane involves the interaction of tau with specific lipids, such as PI(4,5)P_2_ (not shown), at the inner leaflet of the plasma membrane and interaction with HSPGs at the outer leaflet of the plasma membrane that facilitates the release of tau to the extracellular space. (2) Exosomal secretion involves tau uptake into intraluminal vesicles (ILVs) of LE/Multivesicular bodies (MVBs) by their inward budding and subsequent release of these ILV (now termed exosomes) *via* fusion of LE/MVBs with the plasma membrane. (3) Autophagy-based secretion involves sequestration of tau by an expanding phagophore and fusion of the resulting autophagosome with the plasma membrane to release tau. Autophagosome may also first fuse with LE/MVB on its pathway to the plasma membrane. (4) LE/lysosome-mediated secretion (misfolding-associated protein secretion, MAPS) involves the capture of tau by USP19 at the ER membrane, subsequent translocation of tau into the lumen of closely contacting LE/lysosomes, facilitated by Hsc70 and its LE/Lys chaperone DNAJC5, and fusion of LE/lysosome with the plasma membrane to release vesicle-free tau. (5) Ectosomal secretion involves the budding of tau-containing extracellular vesicles directly from the plasma membrane to release tau inside vesicles, which are typically larger than exosomes and have different membrane compositions. AP, autophagosome; EE, early endosome; HSPG, heparan sulfate proteoglycans; LE, late endosome; MVB, multivesicular body.

Tau being a cytosolic leaderless protein, normally bound to microtubules is anticipated to follow the unconventional secretory pathways. Indeed, several studies have demonstrated that tau does not follow the ER-Golgi pathway for secretion but multiple unconventional secretory pathways (Figure 1; Chai et al., 2012; Karch et al., 2012; Plouffe et al., 2012; Dujardin et al., 2014; Tang et al., 2015; Fontaine et al., 2016; Katsinelos et al., 2018; Merezhko et al., 2018). Interestingly, it appears that out of these four unconventional secretory pathways for cytosolic proteins, only ABC transporter-based secretion, dedicated to acylated peptides and proteins (Rabouille et al., 2012), has never been shown to mediate tau secretion. It should be emphasized that so far tau secretion *via* unconventional secretion pathways has been demonstrated only with *in vitro* models. Table 1 summarizes findings from the key studies addressing tau secretion mechanisms.

**Table 1:** Summary of main studies supporting different pathways involved in tau secretion.

Secretory pathway		*In vitro*	*In vivo*	Type of tau	Secreted tau	References
Unconventional protein secretion type I/Translocation through the plasma membrane		Cell lines (Neuro2A, SH-SY5Y, CHO, and HEK293), mouse and rat primary neurons		Wt human tau (0N4R, 2N4R) and E14, endogenous murine tau	Full-length and truncated forms, phosphorylated, mostly small soluble oligomers	Chai et al. (2012), Katsinelos et al. (2018), and Merezhko et al. (2018)
Membranous organelles-based secretion	Exosomal secretion	Cell lines (HEK293, COS7, N2A, and M1C), rat primary neurons	Mouse brain, human CSF	Wt human tau (3R, 0N4R, 1N4R), P301S, 4R domain of human tau ΔK280, endogenous rat tau, and endogenous human tau	Full-length and truncated forms, hypophosphorylated, monomeric and oligomeric, partly insoluble	Saman et al. (2012), Simón et al. (2012), Asai et al. (2015), and Wang et al. (2017)
	Autophagy-based secretion	Cell lines (SH-SY5Y and HEK293), murine primary neurons, human iPSC-derived neurons		Wt human tau (all 6 isoforms), endogenous rat tau, endogenous human tau	Full-length and truncated forms	Tang et al. (2015), Lonati et al. (2018), Kang et al. (2019), and Chen et al. (2020)
	LE/lysosomal secretion	Cell lines (HEK293, M17, and HeLa), rat primary neurons, mouse organotypic brain slices		Wt tau (0N4R and 1N4R), R406W, P301L, and endogenous murine tau	Truncated forms, hypophosphorylated	Fontaine et al. (2016), Rodriguez et al. (2017), and Xu Y. et al. (2018)
Ectosomal secretion		Cell line (N1-E115), rat primary neurons	Rat ISF, human CSF	Wt human tau (1N4R), endogenous rat tau, endogenous human tau	Full-length, C- and N-terminal truncated forms	Dujardin et al. (2014) and Spitzer et al. (2019)

*E14, a pseudo hyperphosphorylated form of tau; wt, wild type; CSF, cerebrospinal fluid; iPSC, induced pluripotent stem cell; ISF, interstitial fluid*.

## Direct Translocation Through the Plasma Membrane

Some cytosolic leaderless proteins translocate directly across the plasma membrane to reach the extracellular space as a free protein. The most studied protein that follows this secretory pathway is fibroblast growth factor 2 (FGF2), a secreted growth factor that lacks a signal peptide (Steringer and Nickel, 2018). FGF2 secretion involves the following key steps: (1) recruitment of FGF2 to the plasma membrane by interaction with PI(4,5)P_2_ and the Na/K-ATPase (ATP1A1; Temmerman et al., 2008; Zacherl et al., 2015); (2) phosphorylation of FGF2 by the Tec-kinase (Ebert et al., 2010); (3) PI(4,5)P_2_-dependent oligomerization of FGF2 and membrane pore formation (Steringer et al., 2012); and (4) binding to heparan-sulfate chains of cell surface HSPGs resulting in translocation of FGF2 molecules to the extracellular side (Zehe et al., 2006; Nickel, 2007; Steringer and Nickel, 2018). Another well-studied protein that follows this secretory pathway is the human immunodeficiency virus type 1 transactivator of transcription (HIV-Tat), whose secretion is similar to FGF2 with some distinctive features (Rayne et al., 2010; Zeitler et al., 2015; Agostini et al., 2017). Recent studies in immortal cell lines and rodent neuronal cultures suggest that another protein that can follow this unconventional secretory pathway is tau (Katsinelos et al., 2018; Merezhko et al., 2018).

The first hint for direct secretion came from the observation that the majority of tau in the extracellular media or human CSF appears as vesicle-free protein (Chai et al., 2012; Karch et al., 2012; Plouffe et al., 2012; Dujardin et al., 2014; Yan et al., 2016; Wang et al., 2017; Guix et al., 2018; Katsinelos et al., 2018). Besides translocation through the plasma membrane, however, free tau may originate from autophagy-based secretion and LE/lysosomal secretion, two sub-types of MOBS. Tau secretion, however, was shown to be independent of Ca^2+^ and ATP, strongly suggesting that the process does not involve membrane fusion events in these cells, which excludes MOBS (Karch et al., 2012; Merezhko et al., 2018). Further, tau was not found to localize in vesicles, multivesicular bodies (MVBs), or any other intracellular vesicular compartments in cultured cells and was found only in minute quantities in exosomal media fraction produced by tau-expressing cells (Katsinelos et al., 2018; Merezhko et al., 2018). Tau localization at the plasma membrane, on the other hand, has been commonly observed.

The further similarities between the secretion of FGF2 and tau all point out to the ability of tau to follow an FGF2-like secretion pathway. First, tau secretion, as well as FGF2 secretion, involves PI(4,5)P_2_ (Katsinelos et al., 2018). Katsinelos et al. (2018) demonstrated that tau could bind to immobilized PI(4,5)P_2_ and this binding seemed to be required for binding of tau to the membrane. Furthermore, treatment with an antibiotic that blocks the interaction of proteins with phosphoinositides suppressed secretion of phosphomimetic mutant of tau in cultured cells (Katsinelos et al., 2018). Tau binding to the plasma membrane, however, may not solely rely on PI(4,5)P_2_, as is the case with FGF2, but resembles HIV-Tat protein, which can bind to a range of acidic membrane lipids at the inner leaflet of the plasma membrane (Temmerman et al., 2008; Zeitler et al., 2015; Katsinelos et al., 2018).

Binding of FGF2 to PI(4,5P)_2_ seems to be facilitated by cholesterol and sphingomyelin, although both lipids are not essential for the process (Temmerman et al., 2008; Steringer et al., 2017). Accordingly, both cholesterol and sphingomyelin also support the secretion of tau (Merezhko et al., 2018). Cholesterol and sphingomyelin may exert their effect *via* the formation of cholesterol/sphingomyelin-rich microdomains that cluster together molecular components required for FGF2 and tau secretion such as PI(4,5)P_2_ (Pike and Miller, 1998; Johnson et al., 2008). Indeed, several studies have reported that tau localizes to cholesterol/sphingomyelin-rich membrane microdomains in the brain of AD patients, mouse models of AD, and in cell culture (Kawarabayashi et al., 2004; Hernandez et al., 2009; Collin et al., 2014). Furthermore, tau was shown to localize to the plasma membrane in small clusters, which could represent cholesterol/sphingomyelin-rich microdomains (Merezhko et al., 2018).

Second, the secretion of both FGF2 and tau involves phosphorylation (Plouffe et al., 2012; Katsinelos et al., 2018; Merezhko et al., 2018). Although FGF2 secretion requires specific phosphorylation by the Tec-kinase at tyrosine 82, phosphorylation at multiple sites seems to drive tau secretion (Ebert et al., 2010; Plouffe et al., 2012; Katsinelos et al., 2018; Merezhko et al., 2018). Accordingly, as compared to wild-type tau, the expression of a phosphomimetic mutant of tau resulted in a higher level of tau detected at the extracellular side of the plasma membrane and in the extracellular media (Plouffe et al., 2012; Katsinelos et al., 2018).

Third, tau may undergo secretion *via* the formation of a transient membrane pore similar to FGF2 (Steringer et al., 2012). Tau has been shown to disrupt membranes by forming pore-like amyloid structures, and post-translational modifications and pathological mutations enhanced this process (Lasagna-Reeves et al., 2014; Patel et al., 2015). Katsinelos et al. (2018) also reported that tau binding to PI(4,5)P_2_-containing LUVs was accompanied by local disruption of the membrane integrity. Additionally, tau secretion may involve the formation of intermolecular disulfide bridges as occurs with FGF2 secretion (Müller et al., 2015). Indeed, two cysteine residues located in the R2 and R3 microtubule-binding repeats of tau are known to form intermolecular disulfide bridges in tau dimerization and disulfide cross-linked tau dimer were shown to have higher seeding efficiency (Bhattacharya et al., 2001; Kim et al., 2015). The requirement of disulfide bridge formation in tau secretion, however, remains to be demonstrated.

Lastly, similarly to FGF2, tau secretion involves HSPGs on the cell surface, as a decrease in cell surface HSPG level effectively suppressed tau secretion, which was also observed in a cell line deficient in proteoglycan biosynthesis (Katsinelos et al., 2018; Merezhko et al., 2018). Interestingly, even the presence of HSPG on neighboring cells was sufficient to facilitate tau secretion (Katsinelos et al., 2018). As tau can bind to HSPG polysaccharides *in vitro* (Goedert et al., 1996; Hasegawa et al., 1997), it seems plausible that HSPG on the extracellular side of the plasma membrane could bind to the membrane-embedded tau and facilitate its extraction to the extracellular space, similar to FGF2 secretion process (Steringer and Nickel, 2018).

Importantly, tau secreted *via* membrane translocation can enter recipient cells and induce tau aggregation, implying the potential role of this secretory mechanism in tau propagation (Katsinelos et al., 2018; Merezhko et al., 2018).

## Membranous Organelle-Based Secretion (MOBS)

Although MOBS comprises several mechanisms, all of them follow the same basic principle. First, the secreted cytosolic protein enters a carrier organelle, which subsequently fuses with the plasma membrane, releasing its content into extracellular space (Figure 1, pathways 2–4).

Cytosolic proteins can be taken up into intraluminal vesicles (ILVs) by inward budding of late endosomes (LE)/MVBs, which may then fuse with the plasma membrane to release the ILVs to the extracellular space (the secreted ILVs are called exosomes; Nickel and Rabouille, 2009; Rabouille, 2017). Another option for a cytosolic protein is to enter the lumen of a LE/lysosome (Rabouille, 2017; Lee and Ye, 2018). Alternatively, an expanding phagophore may sequester cytosolic proteins, leading to the formation of an autophagosome, which fuses with the plasma membrane instead of a lysosome, also releasing its content to the extracellular space (Claude-Taupin et al., 2017; Rabouille, 2017). Cytosolic proteins may also enter the lumen of phagophores instead of their cytosolic interior and therefore occupy space between the autophagosome outer and inner membranes (Zhang et al., 2015).

Therefore, at least three MOBS sub-pathways exist: exosome-based, autophagy-based, and LE/lysosome-based secretion pathways (Rabouille, 2017; Lee and Ye, 2018). Although it seems that such separation mainly originates from an organelle that fuses with the plasma membrane, the actual difference is the organelle entry mechanism for cytosolic proteins. Thus, exosomal or MVB-based secretion implies the entry of the cytosolic protein into ILVs during the process of inward budding, while LE/lysosomal secretion implies the entry to the lumen of LE/lysosome directly through its membrane, and autophagosome-based secretion—through the formation of a phagophore. The entry mechanism also determines if the contents are secreted in a free form or inside vesicles.

These sub-pathways, however, do not function independently but rather co-operate dynamically, potentially releasing a mixed population of cargo that has entered the organelles by alternative pathways, which creates challenges in their mechanistic investigation (Lee and Ye, 2018; Xu J. et al., 2018). For example, autophagosomes can fuse with LE/MVBs, or LE/MVBs may contain not only ILVs formed by inward budding but also free luminal proteins that translocated through its membrane (Claude-Taupin et al., 2017; Lee and Ye, 2018; Xu J. et al., 2018). In these two cases, the fusion of the organelles with the plasma membrane may release the secretory proteins both inside the vesicles and in a free form.

The endosomal-lysosomal pathway is of great interest in the context of tau secretion, as it is closely related to MOBS. The endosomal-lysosomal pathway comprises several very dynamic compartments, undergoing continuous transformation and exchange of materials. These compartments are early endosome (EE), MVB, LE, and lysosome.

EE, the first organelle in the endosomal-lysosomal pathway, receives the cargo material *via* fusion with multiple endocytic vesicles from several internalization routes and sorts the cargo between tubular extensions and cisternal regions to forward them for recycling to the plasma membrane and degradation pathways, respectively (Jovic et al., 2010; Naslavsky and Caplan, 2018). The membrane in the cisternal region of EE buds inward generating ILVs comprising membrane cargo that have to be degraded (Scott et al., 2014). This part of EE detaches, or matures, from the EE and becomes an MVB (Scott et al., 2014).

MVBs, LEs, and lysosomes are part of a continuum of the endolysosomal system and can be difficult to distinguish when defining a secretion mechanism. They share many markers complicating their identification by immunochemical methods. Different cell types may also present these organelles very differently. Nevertheless, the transition from EE to MVBs, LEs, and lysosomes involve the decrease in luminal pH due to activity of vacuolar H^+^-ATPase (V-ATPase), generation of additional ILVs, change of membrane proteins and lipids, and acquisition of acid hydrolases (Huotari and Helenius, 2011). For this review, we will use terms MVBs and LEs interchangeably and the term LE/lysosome to describe the degradative compartment including LE, lysosomes, and organelles with characteristics of both.

### Exosome-Based Secretion

Inward budding of the endosomal membrane captures cytosolic proteins and traps them inside of ILVs. Forming ILV can sequester a random portion of the cytosol, thus acquiring material unselectively, or work with high precision to acquire specific molecules (Sahu et al., 2011; Villarroya-Beltri et al., 2014). For example, cytosolic proteins containing KFERQ sequence motif are selectively delivered into the forming ILVs by the cooperation of the cytosolic chaperone Hsc70 and endosomal sorting complexes required for transport (ESCRTs), which play an important role in the formation of these vesicles (Sahu et al., 2011; Hurley, 2015). Naturally, not only cytosolic proteins are selectively acquired by forming ILVs, but also membrane proteins, lipids, and RNAs.

The resulting MVBs would either fuse with a lysosome to degrade their contents, or fuse with the plasma membrane and release the ILVs into the extracellular space. The mechanism that regulates the “fusion fate” of MVBs is not clear. One possibility is the existence of special populations of MVBs that can fuse with the plasma membrane; these populations may arise from different sorting mechanisms, resulting in distinct cargo inside of ILVs and the distinct fate of MVBs. For instance, sorting of proteins for degradation may occur *via* ESCRT-dependent ILVs, giving rise to a population of MVBs that fuses with lysosomes; while sorting of proteins for secretion may occur *via* lipid microdomain-dependent ILVs, giving rise to a population of MVBs that fuses with the plasma membrane (Trajkovic et al., 2008). Furthermore, the lipid microdomains on the membrane of MVBs may also control their fusion with the plasma membrane, thus providing secretion of specific cargo (Möbius et al., 2002). Nevertheless, MVBs aimed for secretion translocate along microtubules, dock at the plasma membrane, and undergo Ca^2+^-regulated fusion, which is dependent on soluble *N*-ethylmaleimide-sensitive factor attachment protein receptors (SNAREs), providing multiple additional levels of regulation of exosomal secretion (Hessvik and Llorente, 2018).

Exosome-dependent secretion is the first proposed and perhaps the most studied mechanism for tau secretion (Saman et al., 2012). Endogenous and overexpressed tau have been detected in exosomes from immortal cell lines, rodent neuronal cultures, human iPSC neurons, mouse brain, and from CSF of AD patients (Saman et al., 2012; Dujardin et al., 2014; Tang et al., 2015; Polanco et al., 2016; Wang et al., 2017; Guix et al., 2018). Conversely, a large number of studies failed to detect either endogenous or overexpressed tau in exosomes from the culture media of cell lines or rodent primary neurons (Fauré et al., 2006; Chai et al., 2012; Karch et al., 2012; Plouffe et al., 2012; Santa-Maria et al., 2012). Other studies, however, reported a presence of a small pool of extracellular tau inside the vesicles (generally less than 10% of total extracellular tau) from both cell and animal models, and human CSF (Chai et al., 2012; Wegmann et al., 2016; Yan et al., 2016; Wang et al., 2017; Guix et al., 2018; Katsinelos et al., 2018).

Although the physiological secretion of tau may occur *via* the exosomal pathway, multiple studies emphasize the importance of exosomal secretion in the propagation of tau pathology. First, several recent studies have indicated that the exosomal secretion mechanism contributes to tau secretion more extensively in pathological conditions (Dujardin et al., 2014; Fiandaca et al., 2015; Winston et al., 2016; Wang et al., 2017). Furthermore, exosomes from the CSF of AD patients or controls contained a higher percentage of oligomerized tau than a non-exosomal portion (Wang et al., 2017). Additionally, neuronally derived extracellular vesicles (NDEVs) from patients with mild cognitive impairment (MCI), AD and AD model systems were able to deliver pathological tau to unaffected cells, seed tau aggregation in cell culture and in wild type mice, and induce long-distance propagation of tau pathology (Baker et al., 2016; Polanco et al., 2016; Winston et al., 2016, 2019; Wang et al., 2017). In addition to that, uptake and seeding of aggregation occurred with higher efficiency with exosomal tau as compared with free tau (Asai et al., 2015; Yan et al., 2016; Wang et al., 2017). Finally, inhibition of exosome formation blocked tau propagation in a mouse model (Asai et al., 2015). Thus, although the exosomal pathway contributes to the secretion of only a minor portion of tau, it still may play an important role in the propagation of the pathology.

### Autophagy-Based Secretion

Macroautophagy (from here referred to as autophagy) is a lysosomal degradative pathway in which double-membrane structures, called autophagosomes, isolate cytosolic constituents and typically deliver them to lysosomes for degradation by lysosomal enzymes in a resulting organelle called autolysosome (Dikic and Elazar, 2018). Alternatively, autophagosomes may first fuse with MVBs to form the amphisomes, which subsequently fuse with lysosomes to finally degrade the cargo (Berg et al., 1998). Nonselective degradation of cytosolic material by autophagy provides nutrients to maintain cellular homeostasis and protect the cell from damage under various conditions of cellular stress, such as amino acid or energy shortage (Dikic and Elazar, 2018). Autophagy also protects cells from harmful cytosolic material, such as protein aggregates or damaged organelles, by highly selective elimination of these structures (Dikic and Elazar, 2018).

Degradation of the captured material, however, is not the only possible outcome of autophagy. Instead of fusion with lysosomes, autophagosomes may release their content to the extracellular space by fusion with the plasma membrane (Claude-Taupin et al., 2017). Among the known cargo of secretory autophagy in mammals are phospholipid-binding protein Annexin A2 (ANXA2), aggregation-prone α-syn, and the most studied protein in the context of secretory autophagy in mammals—the proinflammatory cytokine IL-1β (Dupont et al., 2011; Ejlerskov et al., 2013; Öhman et al., 2014; Chen et al., 2017; Minakaki et al., 2018).

An intriguing question in the field of secretory autophagy is when and how the two autophagy pathways diverge. The precise mechanism that separates these pathways is still rather elusive, but several recent articles are starting to shape our understanding of the machinery that mediates secretory autophagy. It appears that the differences may originate at the stage of the cargo selection in the expanding phagophore. In selective autophagy, unique or tag-specific autophagy receptors simultaneously bind the cargo material and autophagy-related protein 8 (ATG8)-family proteins on the phagophore membrane, thus tethering the cargo to the phagophore (Zaffagnini and Martens, 2016). Kimura et al. (2017) have recently identified the first autophagy receptor specific for the secretory autophagy of IL-1β in response to lysosomal damage—TRIM16 (Kimura et al., 2017). This receptor tethers activated IL-1β and result in different utilization of the SNAREs by an autophagosome to mediate the fusion with the plasma membrane instead of a lysosome.

Although multiple aspects of the secretory autophagy remain unclear, several recent articles imply that tau may also follow this secretory pathway. Indeed, the accumulation of autophagosomes and the presence of tau in these structures is a prominent feature in AD and other tauopathies, so it would not be surprising if some of these structures would be redirected for secretion (Piras et al., 2016). Furthermore, a recent study presented electron microscopy images of presumably tau-containing autophagic vacuoles approaching the plasma membrane and possibly releasing free tau in neuroblastoma cells (Tang et al., 2015).

Accumulation of autophagosomes that can be redirected for secretion in tauopathies may arise from enhanced induction of autophagy, impaired lysosomal clearance, or a combination of both. In several studies in neuroblastoma cells and rodent primary neuron cultures, induction of autophagy by either starvation or pharmacological agents was shown to enhance tau secretion, while inhibition of autophagy suppressed it (Mohamed et al., 2014; Lonati et al., 2018; Kang et al., 2019). In other studies, however, the inverse relationship between the induction of autophagy and tau suppression was observed (Tang et al., 2015; Chen et al., 2020). The conflicting results might arise from the dual role of autophagy in tau pathophysiology. Although in any case induction of autophagy results in autophagosome accumulation, the organelles may be directed for degradation or secretion, yielding opposing results on tau secretion. For instance, in one of the studies, where induction of autophagy by oxygen-glucose deprivation in rodent neuronal culture resulted in elevated tau secretion, activation of caspase-3 was reported, which redirects autophagy from degradation to secretion (Sirois et al., 2012; Lonati et al., 2018).

The accumulation of autophagosomes apparent in tauopathies may result not only from increased induction of autophagy but also from impaired lysosomal degradation. Indeed, impairing autophagosome-lysosome fusion or inhibition of lysosomal function both enhanced tau secretion, as was the case for the α-syn (Ejlerskov et al., 2013; Mohamed et al., 2014; Kang et al., 2019; Chen et al., 2020). Such manipulations, however, are not that straightforward to interpret, as they may also affect lysosomal secretion.

Interestingly, amphisomes can also secrete their content upon fusion with the plasma membrane and it appears that both tau and α-syn may undergo secretion in these organelles (Claude-Taupin et al., 2017; Lonati et al., 2018; Minakaki et al., 2018). Lonati et al. (2018) demonstrated that induction of autophagy by starvation, though enhanced secretion of free tau, elevated the secretion of vesicular tau to a greater extend. They also demonstrated the increase of autophagosome marker LC3 in these extracellular vesicles, which implies the possibility of tau secretion *via* amphisomes. Although it is also possible that the majority of tau may arrive in amphisomes *via* MVBs instead of autophagosomes since starvation is known to direct MVBs from exosomal release to fusion with autophagic vacuole to form amphisomes (Fader et al., 2008).

Currently, it is still unclear if tau uses autophagosomes as a carrier in the secretion process. Factors that complicate interpretation of this data include: (1) localization of tau in autophagosomes and amphisomes could simply reflect tau degradation by autophagy; (2) altering the level of autophagy-related proteins may have an impact on other processes in cells; and (3) close connection between autophagy and other pathways of unconventional secretion (Xu J. et al., 2018).

### Late Endosomal/Lysosomal Secretion

In some cells (including immune cells, astrocytes, and catecholaminergic neurons) specialized secretory lysosomes (called lysosome-related organelles) execute regulated secretion (Luzio et al., 2014). Conventional LEs/lysosomes in other cell types, however, are also able to secrete their content (Luzio et al., 2014; Lee and Ye, 2018). Besides having a role in unconventional protein secretion, this process is also essential for plasma membrane repair (Samie and Xu, 2014).

Chaperone-mediated autophagy (CMA) delivers cytosolic proteins into the LE/lysosomes (Lee and Ye, 2018). Regularly, CMA involves the recognition of cytosolic proteins containing a KFERQ-like motif by heat shock cognate 71 kDa protein (Hsc70) and its co-chaperones, followed by unfolding-coupled translocation to the lumen of LE/lysosome mediated by LAMP2A protein on the membrane of this organelle (Kaushik et al., 2011). LE/lysosomal secretion of misfolded proteins involves a similar organelle entry mechanism, which is called the misfolding-associated protein secretion pathway (MAPS). During cellular stress and overloading of the ubiquitin-proteasome system, MAPS functions to translocate misfolded cytosolic proteins, including tau and other neurodegeneration disease-associated proteins, to LEs/lysosomes for secretion to extracellular space (Fontaine et al., 2016; Lee et al., 2016). In this pathway, ubiquitin carboxyl-terminal hydrolase 19 (USP19) uses its chaperone activity to capture a misfolded cytosolic protein at the extracellular surface of the ER (Lee et al., 2016). Following capture, Hsc70 and it’s co-chaperone DnaJ heat shock protein family member C5 (DNAJC5) translocate the cargo protein into the lumen of LE/lysosome that associates with ER; in this process, DNAJC5 translocates into the lumen of LE/lysosome and undergoes secretion together with a cargo protein (Xu Y. et al., 2018).

Although this mechanism reminds CMA as it also utilizes Hsc70 and involves a protein translocation through the LE/lysosomal membrane, MAPS is very different (Lee and Ye, 2018). CMA pathway captures proteins containing the KFERQ-like motif and requires unfolding during translocation, while the MAPS pathway is specific for misfolded proteins and can translocate them to LE/lysosome without unfolding (Chiang and Dice, 1988; Lee et al., 2018). These pathways also have opposing regulatory mechanisms; for example, cellular starvation activates CMA but inhibits the MAPS pathway (Cuervo et al., 1995; Lee et al., 2018).

Besides cargo loading, LE/lysosomal secretion involves three additional steps, the same as in exosomal secretion. First, upon stimulation LEs/lysosomes are transported along the microtubules from perinuclear and cytosolic areas to the cell periphery; this translocation changes the properties of these organelles (Spampanato et al., 2013; Johnson et al., 2016; Pu et al., 2016). Peripheral LEs/lysosomes are less acidic, have impaired proteolytic activity, and have a slightly different composition, compared to perinuclear ones (Johnson et al., 2016). Following tethering, LEs/lysosomes are tightly docked to the plasma membrane by the preassembly of the SNARE complex, consisting of LE/lysosomal VAMP7 or VAMP8 and the plasma membrane SNAP23 and possibly syntaxin-4 (Rao et al., 2004; Samie and Xu, 2014; Fontaine et al., 2016; Lee et al., 2016). In the last stage, LE/lysosomes fuse with the plasma membrane; the fusion is triggered by a localized rise in intracellular Ca^2+^ level (Rodríguez et al., 1997; Reddy et al., 2001; Savina et al., 2003; Liu et al., 2011; Medina et al., 2011).

Originally, two publications suggested that tau can undergo secretion *via* the MAPS pathway at least in various *in vitro* models (see Table 1, Fontaine et al., 2016; Xu Y. et al., 2018). In 2016, Fontaine and colleagues (Fontaine et al., 2016) described a mechanism of tau secretion that depends on DNAJC5-Hsc70 complex and involves encapsulation of cargo to an unidentified membranous compartment that fuses with the plasma membrane. At the same time, Lee et al. (2016) described a USP19-dependent mechanism for the selective secretion of misfolded proteins and named this pathway the MAPS (Lee et al., 2016). Only the extensive results obtained by Xu Y. et al. (2018) 2 years later, however, allowed to realize that the mechanism described by Fontaine is MAPS and that tau is one of the MAPS cargos. Additional support for these findings came from Rodríguez et al. (1997), showing that Rab7 GTPase positively regulates the secretion of free tau, suggesting the contribution of LE/lysosomes in the secretion as Rab7 is essential for their maturation and trafficking (Rodriguez et al., 2017). Nevertheless, the role of LE/lysosomes in tau secretion remains poorly understood and further studies are required to shed more light on the mechanisms of the MAPS pathway.

Interestingly, a recent study by Wiersma et al. (2019) may explain why some LEs/lysosomes are rerouted for exocytosis in tauopathies. In this study, seeding of tau pathology induced the formation of LE/lysosomal structures with a dense core consisting of degraded endocytic material, called granulovacuolar degeneration bodies (Wiersma et al., 2019). As these structures seem to appear in neurons at early stages of tau pathology development, before the formation of tau aggregates, it may suggest that LE/lysosomes are facing a shortage of lysosomal enzymes when attempting to degrade early pathogenic tau species (Köhler, 2016). To eliminate tau in such circumstances, LEs/lysosomes may activate the MAPS pathway to compensate for the shortage of lysosomal enzymes. Prior secretion, however, LE/lysosome may truncate tau before its MTBD, making the secreted species seeding-incompetent and, therefore, alleviating intracellular tau pathology and tau spreading in the same time (Xu et al., 2020).

## Ectosome Shedding at the Plasma Membrane

Exosomes are not the only type of extracellular vesicles that are used for the secretion of cytosolic leaderless proteins like tau. The other type is called ectosomes (or microvesicles, shedding vesicles). Compared to exosomes, they are bigger (with a diameter of 100–1,000 nm vs. 30–150 nm of exosomes), have a more irregular shape, and have different membrane composition (Barteneva et al., 2013; Kalra et al., 2016). Ectosomes also have a different origin—they bud outward directly from the plasma membrane. Although the membrane of ectosomes is formed from the plasma membrane, its composition can differ considerably owing to the sorting mechanism involved in ectosome formation (Pollet et al., 2018). Ectosomes, as well as exosomes, are very heterogeneous: not only different cell types shed ectosomes *via* distinct although poorly understood mechanisms, but different mechanisms of the ectosome formation may function either simultaneously or sequentially inside a single cell under different conditions or stimuli (Meldolesi, 2018).

Formation of ectosomes begins with two primary events: an increase in Ca^2+^ concentration and local rearrangement of plasma membrane lipids and proteins to form membrane microdomains (van Niel et al., 2018). These events result in a local loss of interaction between the plasma membrane and cortical actin cytoskeleton and partial disintegration of the cytoskeleton mostly by Ca^2+^-dependent protein degrading enzymes such as calpains (Yano et al., 1993; Pasquet et al., 1996; Kalra et al., 2016; Taylor et al., 2017). Additionally, Ca^2+^-dependent lipid translocases generate local alterations in the distribution of phospholipids of the plasma membrane (van Niel et al., 2018). In particular, phosphatidylserine (PS) and phosphatidylethanolamine (PE), which are normally actively sequestered to the inner leaflet of the plasma membrane, translocate to the outer leaflet (van Meer et al., 2008; van Niel et al., 2018). Exposure of PS to the outer leaflet of the plasma membrane causes membrane bending and further disintegration of the cytoskeleton, enhancing ectosome budding (Kalra et al., 2016). The resulting ectosomes have PS on the surface, which allow their identification *via* probes conjugated to PS-binding proteins, such as Annexin V (Wang et al., 2015). The clustering of cholesterol, sphingomyelin, and ceramide, the product of sphingomyelin hydrolysis, at the plasma membrane facilitates the externalization of PS and membrane budding (Kunzelmann-Marche et al., 2002; Bianco et al., 2009; Pollet et al., 2018).

Stabilization of phospholipid asymmetry, however, failed to prevent Ca^2+^-induced ectosome formation, suggesting the existence of alternative mechanisms (Bucki et al., 1998). The latter is also supported by the finding of ectosomes that do not have PS on their outer leaflet (Elliott et al., 2006; Connor et al., 2010). In the final step, the activation of small GTPase ADP-ribosylation factor 6 (ARF6) initiates a cascade of events, leading to phosphorylation of myosin light chain at the neck of budding vesicles and subsequent actomyosin contraction, resulting in pinching off the ectosome (Muralidharan-Chari et al., 2009; Kalra et al., 2016).

Sorting of cytosolic proteins to ectosomes occurs during their formation when the cytoskeleton disintegrates facilitating vesicle formation and access of cytosolic proteins to the lumen of the newly forming ectosomes (Kalra et al., 2016). The mechanism of this sorting has only recently started to unravel, and currently, there are more questions than answers. Cytoplasmic proteins are sorted to the lumen of budding ectosomes based on their affinity to membrane lipids or proteins, as in sorting to ILVs (van Niel et al., 2018). A portion of cytosolic proteins moves to the lumen of ectosomes arbitrarily since their concentration there resembles one in the cytosol (Meldolesi, 2018). Other proteins, however, are actively sorted in the lumen of ectosomes, which is suggested by their enrichment in these organelles. The sorting may depend on the unspecific interactions with the plasma membrane or specific sequence motifs. Fang et al. (2007) demonstrated that plasma membrane-anchoring and higher-order oligomerization target cytosolic proteins to ectosomes (Fang et al., 2007; Shen et al., 2011). Alternatively, proteins may enter the ectosome lumen *via* interaction with transmembrane proteins or anchored complexes (Yang and Gould, 2013).

All cell types can shed ectosomes under suitable stimulation; the rate of shedding and type of stimuli inducing shedding, however, varies considerably between cell types (Cocucci and Meldolesi, 2015; Meldolesi, 2018). Induction of ectosome shedding can result from activation of P_2_X_7_ receptor (by an agonist or extracellular ATP), activation of PKC, an increase in cytosolic free Ca^2+^, and from depolarization in neuronal cells (Cocucci and Meldolesi, 2015; Toki et al., 2015; Meldolesi, 2018). Most likely, ectosomal shedding is a limited process as it requires significant membrane turnover.

Ectosomal secretion of tau is a poorly studied process with only a few publications existing to date. This mechanism of secretion, however, seems very plausible for tau. First, tau has been detected in ectosomes purified from multiple systems, including neuroblastoma cells, primary cortical neurons, mouse ISF, and cerebrospinal fluid of AD patients and healthy controls (Dujardin et al., 2014; Spitzer et al., 2019). Second, tau possesses two capabilities of proteins enriched in the lumen of ectosomes: tau can (1) bind to the plasma membrane; and (2) form higher-order oligomers (Fang et al., 2007; van Niel et al., 2018). Interestingly, also FGF2 seems to be able to escape cells in ectosomes, suggesting that the same mechanisms may support both the membranous pore formation and sorting to ectosomes (Taverna et al., 2003; Schiera et al., 2007; Proia et al., 2008).

Thus, tau is likely secreted *via* two types of vesicles in both health and disease—exosomes and ectosomes. Exosomes are the main type of vesicles for tau secretion in both conditions but in healthy cells as compared to the cells modeling tau pathology a larger proportion of tau is secreted *via* ectosomes (Dujardin et al., 2014; Spitzer et al., 2019).

## The Role of Neuronal Activity in Tau Secretion

Although cell-to-cell transmission of tau does not exclusively rely on synaptic contacts, the formation of such contacts and neuronal activity appears to promote secretion and spreading of tau (Yamada et al., 2014; Calafate et al., 2015; Schultz et al., 2018). While the role of neuronal activity in tau uptake has not been examined, several studies have demonstrated its effect on tau secretion (Pooler et al., 2013; Yamada et al., 2014; Wang et al., 2017).

Neuronal contacts may simply serve as a favorable location for tau secretion due to their unique composition of proteins and lipids. For instance, synapses may concentrate specific HSPGs with a high affinity for tau to facilitate its translocation through the plasma membrane. One of the HSPGs concentrated at synapses is syndecan-2, one of the major syndecans in neurons (Hsueh et al., 1998; Hsueh and Sheng, 1999). Whether this HSPG has a particular role in tau secretion is unknown, but a recent study suggested that all members of the syndecan family can promote tau internalization in cultured cells and therefore may also have a role in its secretion (Hudák et al., 2019). The MAPS co-chaperone DNAJC5 is another protein mediating tau secretion that is abundantly present at presynaptic terminals, where it regulates proteostasis of synaptic proteins together with Hsc70 (Tobaben et al., 2001; Sharma et al., 2011).

Depolarization of the synaptic terminal may further support various mechanisms of unconventional protein secretion. For instance, neuronal activity can enhance tau secretion *via* membranous organelles and ectosomes as these secretion mechanisms would respond to Ca^2+^ influx induced by membrane depolarization at the axon terminal (Rodríguez et al., 1997; Burgoyne and Clague, 2003; Lachenal et al., 2011; Meldolesi, 2018). While the role of neuronal activity in ectosomal tau secretion has not been examined, its positive effect on the secretion of tau inside exosomes has been shown (Wang et al., 2017).

Pooler et al. (2013) however, have demonstrated that neuronal activity promotes largely non-exosomal secretion of tau in primary neurons (Pooler et al., 2013). Furthermore, they showed that tau secretion depended on Ca^2+^ influx following stimulation of AMPA receptors, but only because Ca^2+^ promoted the fusion of synaptic vesicles (SVs) with the plasma membrane upon stimulation. Pooler et al. (2013) concluded that neuronal activity promotes tau secretion *via* a mechanism dependent on SV release—which does not necessarily mean that tau is secreted *via* synaptic vesicles. Although hyperphosphorylated and oligomeric tau indeed binds to the cytosolic side of SVs *via* their transmembrane protein synaptogyrin-3, it is unlikely that tau traverses the membrane of SVs and undergoes release together with neurotransmitters (Zhou et al., 2017; McInnes et al., 2018). Secretion *via* plasma membrane translocation pathway, on the other hand, could explain the effect observed by Pooler et al. (2013).

SVs release neurotransmitters by merging with the synaptic membrane, from where the components of SVs are later retrieved to replenish the pool of SVs by endocytosis (Chanaday et al., 2019). As the protein and lipid compositions of the presynaptic and the SV membranes differ, their fusion may transiently alter the composition of the presynaptic membrane at least in the timespan between fusion and retrieval (Takamori et al., 2006; Lewis et al., 2017). This may make the PM more suitable for tau exit by bringing proteins or lipids favoring tau secretion. As tau associates with SVs, the fusion can also bring the normally cytosolic Tau to the PM (Zhou et al., 2017).

It remains unclear if the components of newly merged SVs largely stay together in clusters or disperse into the synaptic membrane and later regather for retrieval (Willig et al., 2006; Opazo and Rizzoli, 2010; Opazo et al., 2010; Gimber et al., 2015). While post-fusion clustering of SV proteins has been a subject of several studies, if SV lipids remain together on the synaptic membrane remains unclear. Studies in *Drosophila* have shown that recycling and endocytosis of SVs require the presence of sterols that keeps SV components as discrete domains at the synaptic membrane (Dason et al., 2010, 2014). Furthermore, it appears that sterols present in SVs, not the presynaptic membrane, plays a key role in the cycling of SVs (Dason et al., 2010). Thus, it is possible that after SV fusion, tau, arriving at the presynaptic membrane with SVs, becomes associated with cholesterol microdomains enriched with SV components, some of which may favor tau secretion.

## The Role of Glia in Tau Propagation

Recent studies suggest that glial cells are critical players in the tau propagation process (Asai et al., 2015; Narasimhan et al., 2019). Glial cells, consisting of: (1) microglia; (2) astrocytes; (3) oligodendrocytes; and (4) NG2-glia, provide support and protection and therefore are essential for maintaining neuronal functions. Microglia are the resident innate immune cells of the brain involved in the regulation of neuroinflammation, synaptic pruning, and the clearance of microbes, dying cells, and protein aggregates (Colonna and Butovsky, 2017). Astrocytes are complex cells that play diverse supportive functions in the brain including maintenance of electrolyte and lipid homeostasis, uptake and recycling of neurotransmitters, and modulation of synaptic activity (Oksanen et al., 2019). Oligodendrocytes produce the myelin sheath to insulate and metabolically support axons, while NG2-glial cells serve as progenitors for oligodendrocytes in the adult brain to support myelin plasticity (Simons and Nave, 2015; Valny et al., 2017).

Although all types of glial cells are relevant to AD pathogenesis, microglia and astrocytes have received the most attention for their role in mediating neuroinflammation that seems to play an important role in the pathogenesis of AD (Heneka et al., 2015; Kinney et al., 2018). In response to injury or pathological signals, such as Aβ and possibly pathological tau, these glial cells adopt activated phenotype and start releasing inflammatory factors such as cytokines and chemokines (Heneka et al., 2015; Laurent et al., 2018). Although a lot of controversies exist in this research area, it seems that at the early stages of AD, a neuroinflammatory response may be protective, resulting in the efficient elimination of Aβ and pathological tau (Kinney et al., 2018). The continuous activation of glial cells in the course of the disease, however, may lead to the development of dysfunctional chronic inflammation that exacerbates both AD pathologies.

Microglia can eliminate extracellular tau by phagocytizing and degrading it (Asai et al., 2015; Luo et al., 2015; Bolós et al., 2016; Hopp et al., 2018). Indeed, exposure of tau-containing cell lysate to microglia reduced the seeding activity of this lysate, confirming the ability of healthy microglia to degrade seed-competent tau (Hopp et al., 2018). Degradation efficiency, however, may worsen as microglia becomes dystrophic due to the overloading of the clearance system (Vogels et al., 2019). As a result, microglia may enhance tau propagation by secreting tau-containing exosomes, which can propagate the pathology more efficiently than naked pre-aggregated tau (Asai et al., 2015; Hopp et al., 2018). In support of this, the inhibition of exosome biosynthesis or microglia depletion was shown to suppress tau propagation in mouse models of tauopathy and to improve learning and memory in the 3xTg-AD mouse model (APP Swedish, MAPT P301L, and PSEN1 M146V; Asai et al., 2015; Dagher et al., 2015).

If tau phagocytosis by microglia eventually enhances tau propagation, the important question here is where do microglia take tau from? First, microglia may obtain tau by phagocytosis of neurons or synapses containing pathological tau (Brelstaff et al., 2018; Dejanovic et al., 2018). Alternatively, microglia may phagocytize tau secreted to the extracellular space.

As microglia uptakes extracellular vesicles, exosomes can deliver tau to microglia (Paolicelli et al., 2019). The majority of neuronal exosomes secreted upon neuronal stimulation, however, appear to bind specifically to neurons, not to glial cells, suggesting that microglia likely receives the majority of tau not *via* the uptake of tau-containing extracellular vesicles (Chivet et al., 2014). Several studies demonstrated, on the other hand, that microglia can phagocytize free tau, in both soluble and insoluble forms (Asai et al., 2015; Luo et al., 2015; Bolós et al., 2016). Thus, as free tau is present at a relatively high level in the extracellular space and can be internalized to cells, it appears to be a good candidate for the main source of tau “supply” for microglia.

Although less studied, astrocytes may also be a part of the tau propagation process. As astrocytes are well integrated into synapses both physically and functionally, they are well-positioned to interfere with the synaptic transmission of tau. Recent studies suggest that this may indeed be possible (Narasimhan et al., 2017, 2019; Martini-Stoica et al., 2018; Perea et al., 2019). First, astrocytes were shown to uptake tau and suppress its spreading *in vitro*, suggesting a protective role of these cells (Martini-Stoica et al., 2018; Perea et al., 2019). In fact, in cell culture and organotypic brain slices, astrocytes internalized oligomeric tau more efficiently than neurons (Piacentini et al., 2017). It is noteworthy that while uptake of naked monomeric and aggregated tau by cultured astrocytes was observed in several studies, vesicular tau uptake by astrocytes has not been examined (Piacentini et al., 2017; Martini-Stoica et al., 2018; Perea et al., 2019).

Development of astrocytic tau inclusions in multiple tauopathies may also suggest that astrocytes uptake neuronal tau as its expression in these cells is very low (Kahlson and Colodner, 2015). In support, when propagation of neuronal and glial tau pathology was investigated in wild-type mice following the injection of tau extracted from CBD or PSP patient brains to gray matter, astrocytic tau pathology spread into the same brain regions as neuronal pathology (Narasimhan et al., 2017). Furthermore, the intensity of tau pathology in astrocytes negatively correlated with that of neurons, again suggesting uptake of neuronal tau by astrocytes. Finally, when mice with neuron-specific tau knockdown were used for the same experiment, astrocytic tau pathology failed to spread, suggesting that the spread of tau pathology in astrocytes requires uptake of neuronal tau (Narasimhan et al., 2019). It is possible, however, that certain stressors during disease progression could upregulate the expression of tau in astrocytes to the level sufficient to initiate tau pathology.

Irrespective of the origin of tau in astrocytes, an important question is whether astrocytes can pass tau to neurons. Indeed, astrocytes appear to be capable of tau secretion as a recent study demonstrated that in comparison with neuronal exosomes, astrocytic exosomes in human plasma contained several-fold higher levels of hyperphosphorylated tau (Goetzl et al., 2016). Additionally, in certain brain areas of tauopathy patients, the appearance of tau pathology in astrocytes precedes its appearance in neurons (Ling et al., 2016; Kovacs et al., 2020). Furthermore, in ARTAG, tau pathology can be present exclusively in astrocytes (Kovacs et al., 2016). Finally, following injection to wild-type mice brain, tau extracted from brains of ARTAG patients without neuronal tau pathology resulted in a spread of hyperphosphorylated tau in both neurons and glia, suggesting that astrocytic tau seeds are capable of initiating the disease progression (Ferrer et al., 2018). The absence of observable tau pathology in neurons, however, does not mean the absence of pathological neuronal tau able to seed aggregation (DeVos et al., 2018).

In addition to astrocytes, tau-positive inclusions can also appear in oligodendrocytes in tauopathies, and oligodendrocytes do express tau, although at a low level (Kahlson and Colodner, 2015). As with astrocytes, oligodendrocytic tau pathology can spread in the brain of wild-type mice, following the injection of brain extracts from CBD and PSP patients to the gray matter (Narasimhan et al., 2017). In contrast, however, oligodendrocytes were able to propagate CBD oligodendrocytic tau pathology across the brain even in mice with neuron-specific tau knockdown (Narasimhan et al., 2019). Furthermore, oligodendrocytes seemed not to use neurons for the propagation process at all, suggesting a completely independent mechanism of tau propagation in these cells with seeds transferring from one oligodendrocyte to another. Interestingly, injection of tau from AD patients into gray matter failed to result in the spreading of tau pathology in oligodendrocytes (Narasimhan et al., 2017). In contrast, tau injection into the white matter of wild-type mice resulted in tau propagation, even when injected tau was extracted from tauopathies with only neuronal inclusions, AD and PART (Ferrer et al., 2019). Thus, it appears that although oligodendrocytes can spread tau pathology independently, uptake of neuronally secreted tau may trigger the development of such pathology.

## Conclusions

Neurons appear to propagate tau pathology in a variety of ways, which are at least partially modulated by neuronal activity and involve multiple cell types in the brain. Unconventional pathways of tau secretion are likely deeply intertwined with each other, and manipulating one secretory pathway is likely to affect others, complicating mechanistic studies. Naturally, like many processes in neurons, tau secretion does not function independently of neuronal activity. The mechanism of such connection, however, is currently not clear and remains on the most intriguing unanswered questions regarding the tau secretion. Our current understanding of relationships between glial cells and different steps of tau propagation is also limited and would require further *in vivo* research as well as a more complex *in vitro* approaches that could integrate multiple cell types and other contributing factors.

Tau secretion occurs *via* multiple pathways, but it is not clear if the pathways under pathological and physiological conditions are the same or only partially overlapping. Furthermore, from the propagation perspective, not all secreted tau is the same. Tau secreted through different pathways may have different uptake and seeding abilities. Finally, the mechanistic similarities and differences involved in cell-to-cell transmission of different pathologically misfolded proteins require further attention. In summary, the field of pathological propagation of misfolded proteins in neurodegenerative diseases has only recently emerged and will undoubtedly offer many years of exciting research which hopefully, in the end, can be translated to the benefit of numerous patients in need of better treatments.

## Author Contributions

MM, R-LU, and HH contributed to the conceptualization of the article. MM prepared the original draft. MM, R-LU, and HH contributed to review and editing. All authors contributed to the article and approved the submitted version.

## Conflict of Interest

HH is an employee and shareholder of Herantis Pharma Plc, which is not related to this study. The remaining authors declare that the research was conducted in the absence of any commercial or financial relationships that could be construed as a potential conflict of interest.
